# Factors associated with student success in objective structured clinical examinations and written exams

**DOI:** 10.1186/s12909-026-09298-1

**Published:** 2026-05-16

**Authors:** Aurélie Sannier, Guillaume Airagnes, Damien Roux, Alain Cariou, Aurélie Cazes, Viet-Thi Tran, Jennifer Truchot, Martin Flamant, Donia Bouzid, Philippe Ruszniewski, Albert Faye, Natacha Kadlub, Cédric Lemogne

**Affiliations:** 1 Université Paris Cité, UFR de Médecine, Paris, France; 2 Service de Pathologie, AP-HP, Hôpital Bichat, Paris, France; 3 DMU Psychiatrie et Addictologie, AP-HP.Centre, Paris, France; 4 Service de médecine intensive et réanimation, AP-HP, Hôpital Louis Mourier, Colombes, France; 5 Service de médecine intensive et réanimation, AP-HP, Hôpital Cochin, Paris, France; 6 Service d’épidémiologie clinique, Hôpital Hôtel-Dieu, AP-HP, Paris, France; 7 Service d’accueil des urgences, Hôpital Cochin, AP-HP, Paris, France; 8 Service des explorations fonctionnelles multidisciplinaires, Hôpital Bichat, AP-HP, Paris, France; 9 Service d’accueil des urgences, Hôpital Bichat, AP-HP, Paris, France; 10 Service de pancréatologie et oncologie digestive, Hôpital Beaujon, AP-HP, Clichy, France; 11 Service de pédiatrie générale, Hôpital Robert Debré, AP-HP, Paris, France; 12 Service de chirurgie maxillo-faciale pédiatrique, Hôpital Necker, AP-HP, Paris, France; 13 Université Paris Cité and Université Sorbonne Paris Nord, Inserm, INRAE, Center for Research in Epidemiology and Statistics (CRESS), Paris, France; 14 Service de psychiatrie, Hôpital Hôtel-Dieu, AP-HP, Paris, France; 15 Département de Pathologie, Université Paris Cité, AP-HP, Hôpital Bichat, 46 rue Henri Huchard, F-75018 Paris, France

**Keywords:** Medical studies, Objective structured clinical examinations, Written exams, Educational strategies

## Abstract

**Background:**

Objective Structured Clinical Examinations (OSCEs) are an increasingly popular method for assessing medical students, aiming to foster a curriculum that emphasizes the development of clinical skills during medical studies. This study aimed to identify the factors influencing student performance in both OSCEs and written exams to better understand students’ needs and refine educational strategies. We hypothesized that the determinants of success may differ between these two modalities, which evaluate partly distinct competencies.

**Methods:**

A survey was conducted among fifth-year medical students from Université Paris Cité, collecting data on demographics, personal context, educational background, and exam preparation methods. Statistical analyses included univariate and multivariate regression models to examine the association between student characteristics and mean grades in OSCEs and written exams.

**Results:**

The study involved 441 medical students, of whom 310 were women. Factors independently associated with better grades in written exams included the absence of concurrent professional activity, no previous repetition in the curriculum, regular attendance at faculty theory courses, use of reference textbooks, participation in private training conferences, and higher exposure to OSCEs during hospital rotations. For OSCE performance, better grades were independently associated with the subscription to online exam preparation platforms and attendance at private training conferences.

**Conclusions:**

Our findings reveal distinct factors influencing success in OSCEs compared to written exams. Targeted educational strategies, like encouraging OSCE practice during hospital rotations and supporting access to training conferences, may benefit students. A comprehensive understanding of student needs should extend beyond refining teaching strategies to include considerations like scholarship eligibility for students balancing professional work with their studies. These insights highlight the need for a holistic approach in medical education, addressing both academic and personal factors to fully support student achievement.

**Supplementary Information:**

The online version contains supplementary material available at 10.1186/s12909-026-09298-1.

## Background

 Medical studies in France have been reformed to create a curriculum that emphasizes the development of clinical skills, including physical examination, history taking, communication, and procedural abilities, alongside the medical knowledge acquired by students throughout their training. To achieve this goal, the reform of the second cycle of medical studies (i.e., from fourth to sixth year) has introduced new evaluation methods in the sixth year to determine access to residency and specialty choice. Although the French medical studies are characterized by a large part devoted to bedside teaching during hospital rotations, evaluations were so far mainly based on memorization and knowledge restitution in a ranking national exam. The evaluation system now combines written (computerized) exams and Objective Structured Clinical Examinations (OSCEs). All students across the country take the same centrally designed and synchronized examinations. In parallel, medical schools organize local training sessions to prepare students for these national assessments. The written exams focus on medical knowledge and clinical reasoning, integrating diversified approaches like Key-Feature Problems (KFPs) to test knowledge application in clinical scenarios. OSCEs offer a complementary approach by assessing practical skills, including clinical reasoning and communication abilities. OSCEs are used for the assessment of medical students in several countries worldwide [1–6]. Students are placed in a clinical situation with a standardized patient or healthcare professional and evaluating observers. Beyond the objectives that underlie the implementation of these assessment methods, they represent a major challenge for French students as their success in these national evaluations leads to a national ranking upon which their specialization and professional career choice is conditioned.

Previous studies have investigated factors associated with medical students’ performance in both OSCEs and written examinations. In France, studies conducted prior to the recent reform have identified several predictors of success in national ranking examinations, which primarily emphasized memorization and knowledge restitution, including prior academic performance, absence of year repetition, and certain study strategies [7, 8]. Regarding OSCEs, several studies have reported associations between performance and student characteristics such as gender, although findings remain inconsistent across settings [9–11]. Although several studies have reported a positive correlation between OSCE and written examination results, reflecting shared underlying competencies, the strength of this association remains moderate, suggesting that these assessment modalities capture partly distinct dimensions of student performance [12, 13]. Overall, the literature remains limited and heterogeneous, particularly with regard to direct comparisons of factors associated with both OSCE and written examination performance within the same cohort.

In this context, this study aimed to identify the factors associated with student success in these evaluation modalities combining OSCEs and written exams. Given that these two types of assessments evaluate partly distinct competencies, we hypothesized that the determinants of success could differ between them. The results may help better understand student needs and guide educational strategies in medical education.

## Methods

### Study design

This study was conducted among fifth-year undergraduate students at the medical school of Université Paris Cité (Paris, France), representing the first promotion concerned by the new assessment methods associated with the reform of the second cycle. As the study aimed to include the whole cohort of eligible fifth-year students at Université Paris Cité, no a priori sample size calculation or power analysis was performed. A questionnaire was submitted to the students on the examination digital assessment platform during their exams, exam results obtained by the students were also subsequently retrieved. Communication had previously been carried out by e-mail to inform students about the aims of the study and the possibility of completing the survey on the platform during examination sessions. This study obtained the approval of the ethics committee of the Université Paris Cité (2023-SANNIER-EvalR2C). An information note accompanied the survey on examination digital assessment platform to remind students of the aims of the study and to indicate the free and voluntary nature of participation in this project, the processing of personal data and the possibility of withdrawing their consent at any time during the study. The study was reported according to the STROBE (Strengthening the Reporting of Observational Studies in Epidemiology) guidelines, and a completed checklist is provided as supplementary material.

### Data collection

The questionnaire collected general information, including age and gender; personal context, such as concurrent professional activity during medical studies; and educational background, including graduate education prior to medical studies, repeating a year during medical studies outside the first year, and support from university tutoring or mentoring programs. It also collected more specific data on the students’ preparation for the exams, including their attendance at faculty theory courses, number of hours dedicated to the exam preparation each week, sources used by the students for theoretical learning, subscription to online exam preparation websites (fee-based, privately provided digital self-training tools), attendance at private training conferences (fee-based voluntary preparatory courses provided by private organizations outside the university curriculum), group training sessions, completion of OSCEs during hospital rotations (training sessions organized locally within clinical departments), and the results obtained in these evaluations. The questions included in the survey were defined a priori based on existing literature on factors potentially associated with academic performance in medical students, as well as on expert knowledge of the medical curriculum and examination preparation strategies. The full questionnaire is displayed in Appendix 1. Subsequent results obtained by the students during the OSCEs and the faculty written examinations were collected. Two sessions of five stations of 8 min were performed for the OSCEs examination. The written examinations combined various tests, such as progressive clinical case scenarios, KFPs and isolated questions (either multiple-choice questions (MCQs) or short-answer questions). Two grades, each out of 20 points, were calculated for each student participating in the study: one for the OSCEs and one for the written exams.

Questionnaire data were matched with students’ examination results (OSCEs and written exams) using identifiable information provided by the academic administration. This linkage was performed in a secure environment by authorized members of the research team. After matching, the dataset was pseudonymized by assigning a study identifier to each participant and removing all directly identifiable information. The correspondence table between identifiers and personal data was stored separately in a secure location with restricted access. All statistical analyses were conducted using the pseudonymized dataset.

### Statistical analysis

Categorical variables were summarized with raw numbers and percentages. Continuous variables were described using means and standard deviations (SD). OSCE and written exam grades were considered as outcome variables, with the other variables serving as predictors. Associations between grades and categorical variables were analyzed by comparing means with Student’s t-tests, and associations with continuous variables were evaluated using Pearson correlation coefficients. For multivariate analysis, variables associated with written exam grade with a *p*-value < 0.1 in univariate analysis were included in multiple linear regression analyses. While certain factors may seem more relevant to either written exams or OSCEs, all factors were a priori considered for both outcomes. For instance, although the number of OSCEs completed during hospital rotations may seem more directly associated with the OSCE grades, completing OSCEs during hospital rotations may also support clinical skill development, such as clinical reasoning, potentially enhancing written exam performance as well. Two variables were excluded from the multivariate analysis: (1) age, to prevent multicollinearity with the variable indicating year repetition during medical studies, and (2) OSCE results during hospital rotations, due to missing data for 129 students. Results are presented as regression coefficients (β), along with their 95% confidence intervals and *p*-values. A *p*-value < 0.05 was considered significant. Statistical analysis was performed using GraphPad Prism 10 software (GraphPad Software, San Diego, CA).

## Results

### Study population

A total of 441 fifth-year medical students completed the survey (48.3% of the promotion of 913 students), including 310 women (70.3%). The average age of respondents was 23. Eighty-one students (18.4%) had a professional activity concomitant with medical studies. Forty-one respondents (9.3%) pursued graduate education studies prior to medical studies. Forty-two students (9.5%) had repeated a year during their medical studies, apart from the first year. Detailed characteristics of the students included in the study are shown in Table 1.

**Table 1:** Characteristics of fifth-year medical students included in the study

Variables	*N* = 441
Age, mean ± SD, years	23.4 ± 1.9
Female, *n* (%)	310 (70.3%)
Concurrent professional activity, *n* (%)	81 (18.4%)
Prior graduate education, *n* (%)	41 (9.3%)
Repeating a year during medical studies, *n* (%)	42 (9.5%)
Support from the tutoring or mentoring system, *n* (%)	114 (25.9%)
Proportion of faculty theory courses attended	
< 50%	312 (70.7%)
≥ 50%	129 (29.3%)
Hours dedicated to the exam preparation per week, mean ± SD	44.2 ± 15.8
Sources of knowledge used by the students for their theoretical learning, *n* (%)	
Reference textbooks written by the specialty colleges	433 (98.2%)
Books other than the college reference textbooks	98 (22.2%)
Theoretical courses given within the university	218 (49.4%)
Medical revision notes from books or online preparation websites	284 (64.4%)
Video tutorials and podcasts	191 (43.3%)
Subscription to an online exam preparation website, *n* (%)	330 (74.8%)
Attendance at private training conferences, *n* (%)	190 (43.1%)
Group training sessions, *n* (%)	105 (23.8%)
OSCEs during hospital rotations	
0 or 1 in the year, *n* (%)	133 (30.2%)
2 or 3 in the year, *n* (%)	308 (69.8%)
Results in hospital OSCEs, mean ± SD (out of 20 points)	14.9 ± 2.4

*OSCEs* Objective structured clinical examinations

### Factors associated with grades obtained in faculty OSCEs and written exams

The mean grades (+/- SD) obtained by respondents in OSCEs and written exams were 13.16 +/- 1.96 points and 13.85 +/- 1.39 points, respectively. A moderate positive correlation was identified between the mean grade obtained in OSCEs and the mean grade obtained in written exams (*r* = 0.56; *p* < 0.0001). The association between student characteristics and grades is detailed in Table 2.

**Table 2:** Univariate analysis of student characteristics linked to mean OSCE and written exam grades

Categorical variables		OSCEs		**Written exams**	
	*n* (%)	Mean (+/- SD)	*p* value	Mean (+/- SD)	*p* value
Gender					
Female	310 (70.3%)	13.21 +/- 1.84	0.42	13.94 +/- 1.25	0.03
Male	131 (29.7%)	13.05 +/- 2.21		13.63 +/- 1.67	
Concurrent professional activity					
Yes	81 (18.4%)	13.11 +/- 1.99	0.78	13.43 +/- 1.41	< 0.01
No	360 (81.6%)	13.18 +/- 1.96		13.94 +/- 1.37	
Prior graduate education					
Yes	41 (9.3%)	13.35 +/- 1.9	0.52	13.90 +/- 1.35	0.78
No	400 (90.7%)	13.15 +/- 1.96		13.84 +/- 1.4	
Repeating a year					
Yes	42 (9.5%)	12.56 +/- 2.08	0.03	13.02 +/- 1.30	< 0.0001
No	399 (90.5%)	13.23 +/- 1.94		13.93 +/- 1.38	
Tutoring or mentoring system					
Yes	114 (25.9%)	13.29 +/- 1.89	0.43	13.88 +/- 1.33	0.79
No	327 (74.1%)	13.12 +/- 1.98		13.84 +/- 1.42	
Proportion of faculty theory courses attended					
< 50%	129 (29.3%)	13.08 +/- 1.93	0.18	13.72 +/- 1.43	< 0.01
≥ 50%	312 (70.7%)	13.36 +/- 2.00		14.14 +/- 1.24	
Sources of knowledge					
Reference textbooks written by the specialty colleges	404 (91.6%)	13.22 +/- 1.94	0.06	13.90 +/- 1.38	< 0.01
Other sources	37 (8.4%)	12.59 +/- 2.06		13.21 +/- 1.40	
Subscription to an online exam preparation website					
Yes	330 (74.8%)	13.36 +/- 1.85	< 0.001	13.96 +/- 1.35	< 0.01
No	111 (25.2%)	12.57 +/- 2.15		13.51 +/- 1.48	
Attendance at private training conferences					
Yes	190 (43.1%)	13.53 +/- 1.80	< 0.001	14.16 +/- 1.25	< 0.0001
No	251 (56.9%)	12.89 +/- 2.03		13.61 +/- 1.45	
Group training sessions					
Yes	105 (23.8%)	13.35 +/- 1.79	0.26	14.02 +/- 1.28	0.14
No	336 (76.2%)	13.11 +/- 2		13.79 +/- 1.43	
OSCEs during hospital rotations					
0 or 1 in the year	133 (30.2%)	12.92 +/- 1.92	0.08	13.53 +/- 1.43	< 0.01
2 or 3 in the year	308 (69.8%)	13.27 +/- 1.97		13.98 +/- 1.36	

Continuous variables		OSCEs		Written exams	
	Mean (+/- SD)	*r*	*p* value	*r*	*p* value
Age (years)	23.38 +/- 1.94	- 0.06	0.20	- 0.13	< 0.01
Hours dedicated to the exam preparation per week	44.23 +/- 15.78	0.07	0.17	0.08	0.10
Results in hospital OSCEs	14.88 +/- 2.41	0.26	< 0.0001	0.17	< 0.01

*OSCEs* Objective structured clinical examinations

Grades obtained in the written exams were higher among women, students without professional activities outside their medical studies, those who had not repeated a year, attended at least 50% of faculty theory courses, used specialty college reference textbooks as their main knowledge source, subscribed to online exam preparation websites, attended private training conferences, and completed OSCEs in at least two of their three hospital rotations. OSCE grades were higher among students who had not repeated a year, subscribed to an online exam preparation website, and attended private training conferences. No significant differences in mean grades for either written exams or OSCEs were observed based on prior graduate education, participation in university tutoring or mentoring, or attendance at group training sessions. A weak but statistically significant inverse correlation was observed between students’ age and the average grade obtained in written exams (*r* = − 0.13; *p* < 0.01). There was no significant correlation between the number of hours devoted to exam preparation and the result obtained in faculty OSCEs or in written exams.

Factors independently associated with higher grades in written exams included the absence of concurrent professional activities (β = 0.46, 95% CI [0.15; 0.78], *p* < 0.01), no prior repetition during medical studies (β = 0.63, 95% CI [0.20; 1.06], *p* < 0.01), higher attendance at faculty theory courses (β = 0.53, 95% CI [0.26 ; 0.79], *p* < 0.001), use of reference textbooks as the main theoretical source (β = 0.61, 95% CI [0.17 ; 1.06], *p* < 0.01), attendance at private training conferences (β = 0.49, 95% CI [0.23; 0.75], *p* < 0.001), and completing a higher number of OSCEs during hospital rotations (β = 0.4, 95% CI [0.14 ; 0.67], *p* < 0.01). The factors independently associated with a better grade in OSCEs were subscription to an online exam preparation website (β = 0.57, 95% CI [0.14; 1.01], *p* = 0.01) and attendance at private training conferences (β = 0.49, 95% CI [0.11; 0.88], *p* = 0.01). These results are detailed in Table 3.

**Table 3:** Multivariate regression analysis of student characteristics linked to mean OSCE and written exam grades

Student characteristics	OSCEs			Written exams		
	β +/- SD	95% CI	*p* value	β +/- SD	95% CI	*p* value
Female gender	0.03 +/- 0.20	[− 0.37 ; 0.43]	0.90	0.12 +/- 0.14	[− 0.16 ; 0.39]	0.40
No concurrent professional activity	0.02 +/- 0.24	[− 0.45 ; 0.49]	0.93	0.46 +/- 0.16	[0.15 ; 0.78]	< 0.01
No repeating a year	0.47 +/- 0.32	[− 0.17 ; 1.10]	0.15	0.63 +/- 0.22	[0.20 ; 1.06]	< 0.01
Higher proportion of theory courses attended	0.35 +/- 0.20	[− 0.05 ; 0.75]	0.08	0.53 +/- 0.14	[0.26 ; 0.79]	< 0.001
Reference textbooks as the main source of knowledge	0.57 +/- 0.34	[− 0.09 ; 1.23]	0.09	0.61 +/- 0.23	[0.17 ; 1.06]	< 0.01
Subscription to an exam preparation website	0.57 +/- 0.22	[0.14 ; 1.01]	0.01	0.21 +/- 0.15	[− 0.09 ; 0.50]	0.16
Attendance at private training conferences	0.49 +/- 0.19	[0.11 ; 0.88]	0.01	0.49 +/- 0.13	[0.23 ; 0.75]	< 0.001
Higher number of OSCEs during rotations	0.30 +/- 0.20	[− 0.09 ; 0.70]	0.13	0.4 +/- 0.13	[0.14 ; 0.67]	< 0.01
Hours dedicated to the exam preparation per week	0.004 +/- 0.006	[− 0.008 ; 0.020]	0.50	0.005 +/- 0.004	[− 0.003 ; 0.010]	0.25

*OSCEs* Objective structured clinical examinations

## Discussion

The French reform of the second cycle of medical studies (covering the fourth to sixth years) introduced new evaluation methods, combining written exams and OSCEs to enhance clinical skills assessment. The aim of this study was to identify factors associated with student success in these evaluation modalities, providing insights into student needs and informing future teaching strategies.

Our findings reveal distinct factors influencing success in OSCEs compared to written exams. While OSCE success was independently associated with participation in online preparation websites and attendance at private training conferences, success in written exams was linked to a broader range of factors. These included the absence of concurrent professional activities, no prior repetition during medical studies, higher attendance at faculty theory courses, the use of reference textbooks as the main theoretical source, attendance at private training conferences, and completing a higher number of OSCEs during hospital rotations. Some of these factors, such as repeating a year of medical school or attending conferences, have been described in previous studies as associated with ranking in the former French National Ranking, which primarily emphasized memorization and knowledge restitution prior to the implementation of the National Dematerialized Exam and OSCEs [7, 8]. Since OSCEs were introduced to complement written exams, it was expected that grades for these two types of assessments would only show a moderate correlation, as shown in this study and in line with findings from other French or American studies [12, 13]. Therefore, it was also expected that the factors associated with success in each test type would differ. Notably, the two factors independently associated with OSCE performance were not those with the largest effect sizes for written exams, suggesting that these associations are unique to OSCEs rather than weaker reflections of the factors influencing written exams. For instance, online exam preparation websites have incorporated specific tools to help students prepare for OSCEs, potentially aiding those who use them in acquiring the skills required for this evaluation format. Attendance at private training conferences and subscription to online preparation websites may also reflect differences in financial resources, or individual preparation strategies. These variables should therefore be interpreted as potential confounding factors rather than direct effects of the educational tools themselves.

In our study, the mean grade in written exams was significantly higher for women than for men in the univariate analysis, while there was no significant difference in mean OSCE grades between genders. In another study conducted in France on OSCE performance during the second cycle of medical studies, female gender was associated with higher OSCE success. Another French study examining OSCEs as part of the validation procedures to obtain a general medicine diploma also found that women obtained higher average OSCE grades than men [9, 10]. However, these studies examined only factors associated with passing the OSCEs, without evaluating the written test performance of the same students by gender. Several authors, particularly in the United States, have also explored this topic. For example, Bienstock’s study on postgraduate students’ performance in rotation exams found that women outperformed men in both OSCE-type assessments and written tests [11]. These conflicting findings suggest the need for a larger-scale evaluation to determine whether gender might differentially influence success in written exams and OSCEs. Additionally, it would be valuable to assess the correlation between students’ confidence levels and their OSCE performance, incorporating factors that may impact self-confidence, such as gender and social background. A closer examination of the specific skills assessed at each OSCE station is also warranted. For instance, female medical students usually exhibit higher levels of empathy than their male peers, which could affect performance in stations focused on communication skills and may partly explain discrepancies with existing literature [14, 15].

Among the factors not associated with mean OSCE or written exam grades, we note the number of hours dedicated to exam preparation per week, even though this parameter was close to the threshold of statistical significance, with a trend toward a positive correlation with grades. This factor may be challenging for students to accurately estimate, and it also highlights that the quantitative measure of study hours does not necessarily reflect the qualitative aspect of learning, which also occurs daily during clinical rotations.

A key strength of this study is its focus on a substantial sample of 441 students from the first generation of students to be concerned by the new evaluation methods during the sixth year of medical studies in France. However, the response rate of 48.3% raises the possibility of selection bias. Students who participated may have been more engaged in their studies or more interested in exam preparation, potentially limiting the generalizability of the findings. In addition, some variables included in the analysis, such as attendance at private training conferences or engagement in professional activities during medical studies, may reflect underlying social and economic differences between students and could be interpreted as potential confounding factors. Additional limitations include the single-center design and the use of self-reported data for several preparation variables, which may have introduced measurement error or recall bias. Given the observational and cross-sectional design of this study, causal relationships cannot be inferred, and the associations observed should be interpreted with caution. Further research is needed to better understand the causal relationships between preparation strategies and academic performance, particularly through multicenter and longitudinal studies. Exploring the role of socioeconomic factors would also be essential. From an educational perspective, our findings suggest that improving access to structured preparation and supporting students facing financial constraints may help reduce inequalities in academic performance.

## Conclusions

This study identifies key factors associated with student success in OSCEs and written exams, emphasizing the need for targeted educational strategies, such as incorporating regular OSCE practice during hospital rotations and supporting attendance at training conferences. While certain factors, such as concurrent professional activity, cannot be directly influenced, understanding their impact can guide improvements in educational support. These findings underscore the importance of a holistic approach in medical education, extending beyond teaching strategies to include considerations like scholarship eligibility for students balancing professional work with their studies. The study’s insights contribute to refining educational practices and addressing diverse student needs in the evolving landscape of medical training. 

## Supplementary Information

Supplementary Material 1.

Supplementary Material 2.

## Data Availability

The datasets used and analysed during the current study are available from the corresponding author on reasonable request.

## Abbreviations

KFP: Key-feature problem
MCQ: Multiple-choice question
OSCE: Objective structured clinical examination
