# Supplementary material for: Factors associated with student success in objective structured clinical examinations and written exams
Source: BMC Med Educ. 2026 May 16;26:1096. doi: 10.1186/s12909-026-09298-1 (PMC13348646; doi:10.1186/s12909-026-09298-1)
Supplement: Supplementary file 1 — Supplementary Material 1. [file 12909_2026_9298_MOESM1_ESM.docx]

**Questionnaire submitted to students**

How old are you?

Free text

What is your gender?

Male

Female

Do you have a professional activity concomitant with your medical studies?

Yes

No

Did you complete graduate education prior to medical studies?

Yes

No

If so, which one?

Free text

Did you repeat a year during your medical studies (apart from the first year)?

Yes

No

Have you been offered support through the university tutoring or mentoring system?

Yes

No

Do you take part in the university tutoring or mentoring system?

Oui

Non

What proportion of the faculty theory courses do you attend? (give a number between 0 and 100)

Free text

Could you indicate the approximate number of hours of personal work that you dedicate each week to the exam preparation?

Free text

Could you indicate the source(s) of knowledge you use in your theoretical learning? (multiple answers possible)

Reference textbooks written by the specialty colleges

Books other than the college reference manuals

Theoretical courses given within the university

Medical revision notes from books or online preparation sites

Video tutorials and podcasts

Could you identify the main source of knowledge you use in your theoretical learning? (indicate only one answer)

Reference textbooks written by the specialty colleges

Books other than the college reference manuals

Theoretical courses given within the university

Medical revision notes from books or online preparation sites

Video tutorials and podcasts

Do you take part in an online exam preparation site?

Yes

No

Are you attending any private training conference this year?

Yes

No

Do you attend group training sessions?

Yes

No

In which department were you on rotation during the third term?

Free text

Did you have an OSCE test during your third-term rotation?

Yes

No

If so, could you indicate the grade obtained in this third-term rotation OSCE?

Free text

In which department were you on rotation during the second term?

Free text

Did you have an OSCE test during your second-term rotation?

Yes

No

If so, could you indicate the grade obtained in this second-term rotation OSCE?

Free text

In which department were you on rotation during the first term?

Free text

Did you have an OSCE test during your first-term rotation?

Yes

No

If so, could you indicate the grade obtained in this first-term rotation OSCE?

Free text

Do you have any comments on this survey?

Free text
